# Beyond CT: Attenuation correction for stand-alone brain PET^☆^

**DOI:** 10.1016/j.zemedi.2026.02.001

**Published:** 2026-02-04

**Authors:** Markus Jehl, Maria Jose Bonta, Ekaterina Mikhaylova, Max Ahnen, Simon R. Cherry, Ilaria Sacco, Jannis Fischer

**Affiliations:** a Positrigo AG, Switzerland; b Department of Radiology, University of California Davis Health, Sacramento, CA, USA

**Keywords:** Positron emission tomography (PET), Attenuation correction, Neuroimaging, Template registration, Emission-based methods, Deep learning

## Abstract

Accurate attenuation correction (AC) is essential for quantitative brain positron emission tomography (PET), but it presents a significant challenge for dedicated PET-only systems that lack anatomical information from computed tomography (CT) or magnetic resonance imaging. This review provides a comprehensive overview of AC methods designed for stand-alone brain PET. We categorise and analyse existing approaches, including traditional segmentation-based techniques, atlas- and template-driven strategies, methods for simultaneous reconstruction of attenuation and activity, and modern data-driven deep learning techniques. We discuss the inherent trade-offs of each category: template-based methods are computationally efficient and robust for normal anatomy but may fail in atypical cases, while simultaneous reconstruction methods can be powerful but are often sensitive to noise and require time-of-flight information. Deep learning approaches have demonstrated the highest potential for generating patient-specific attenuation maps but are critically dependent on large, diverse training datasets and face challenges in generalisability. Finally, we survey the methods currently implemented in commercial brain-dedicated PET systems, revealing a gap between cutting-edge research and clinical practice, where simpler, more established techniques are still preferred. This review highlights the ongoing need for CT-less AC solutions that balance accuracy, robustness, and practical feasibility for clinical adoption.

## 1 Introduction

Recent advances in molecular neuroimaging and drug development have significantly impacted the diagnosis and management of Alzheimer’s disease. The ability of positron emission tomography (PET) to quantify pathological biomarkers, specifically amyloid and tau proteins, has become central to both research and clinical practice. This has been further emphasised by the regulatory approval of new disease-modifying therapies, such as lecanemab [3] and donanemab [4], which has created a substantial clinical demand for brain PET scans. In response to this need, a number of manufacturers are developing and commercialising dedicated, PET-only brain imaging systems [5], [6], [7], [8], [9], [10], [11].

While these new scanners offer the potential for lower costs and greater patient access, they reintroduce a fundamental challenge in PET imaging: performing accurate attenuation correction (AC) without the anatomical data provided by integrated computed tomography (CT) [12] or magnetic resonance (MR) systems [13], [14].

Accurate attenuation correction is a prerequisite for quantitative analysis in PET, as it accounts for the absorption and scattering of photons within the patient. Historically, this was addressed with transmission scans, which required a ring-shaped or rotating source, longer scans, and additional radiation exposure [15]. This method has largely been replaced by the use of CT or MR data in modern hybrid scanners to generate patient-specific attenuation maps. Standalone PET systems, however, cannot rely on this approach.

Consequently, there is renewed interest in developing and refining AC methods that derive the necessary information from the PET emission signal alone. These techniques vary widely in their approach and complexity. They include iterative algorithms like the maximum likelihood reconstruction of attenuation and activity (MLAA) algorithm [16], data-driven deep learning models that synthesise CT-like attenuation maps or directly reconstruct attenuation corrected images [17], and more straightforward template-based [5] or segmentation-based [6] methods that offer practical advantages for routine clinical workflows.^1^

This paper provides a comprehensive review of these attenuation correction methods that operate without external anatomical data, with a specific focus on their application in brain PET — the primary driver of this recent technological development.

## 2 Attenuation correction

Attenuation is caused by the interaction of the emitted 511 keV photons with the matter they traverse, primarily through Compton scattering, where the photon collides with electrons and loses parts of its energy [18]. Consequently, the attenuation along a given line of response (LOR) is dependent on the distance the photons travel through matter and the density of the material they traverse. The objective of attenuation correction is to account for the loss of these photons. Uncorrected, attenuation produces non-uniform underestimation of radiotracer concentration, particularly in deep or dense tissue, which can distort the appearance of the image. Inaccurate attenuation correction can also lead to an overestimation of radiotracer concentration. Therefore, AC is an essential correction in PET, directly impacting quantification.

**Fig. 1:**
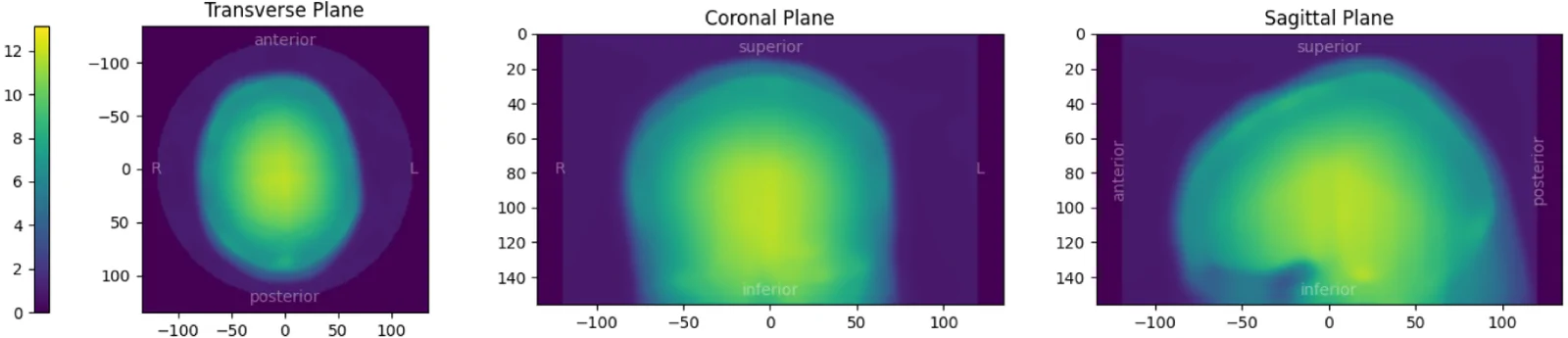
An illustration of head attenuation correction factors. This image was created by reconstructing a simulated phantom of uniform activity while applying a CT-derived attenuation map (inspiration from [19]). This method effectively visualises the correction itself, showing the significant intensity amplification towards the centre of the brain and local corrections for different tissue densities.

Mapping the spatial distribution of attenuation coefficients within the field of view provides attenuation correction factors (ACF) for each LOR, reaching values up to ∼7 for a typical human head. These factors can amplify the reconstructed signal by as much as ∼13 in specific brain regions (Fig. 1). An inaccurate attenuation map (μ-map) can introduce both local errors in the reconstructed image, thereby compromising image interpretation and regional quantitative comparison, as well as a systematic bias in the reconstructed intensity of larger regions, which in turn affects quantitative measures such as standardised uptake value (SUV) or kinetic modelling. The local errors can also propagate non-linearly when quantitative measures employ a reference region. In particular, opposing regional biases can lead to disproportionately large biases in SUV ratios, beyond the magnitude of the individual regional errors.

**Fig. 2:**
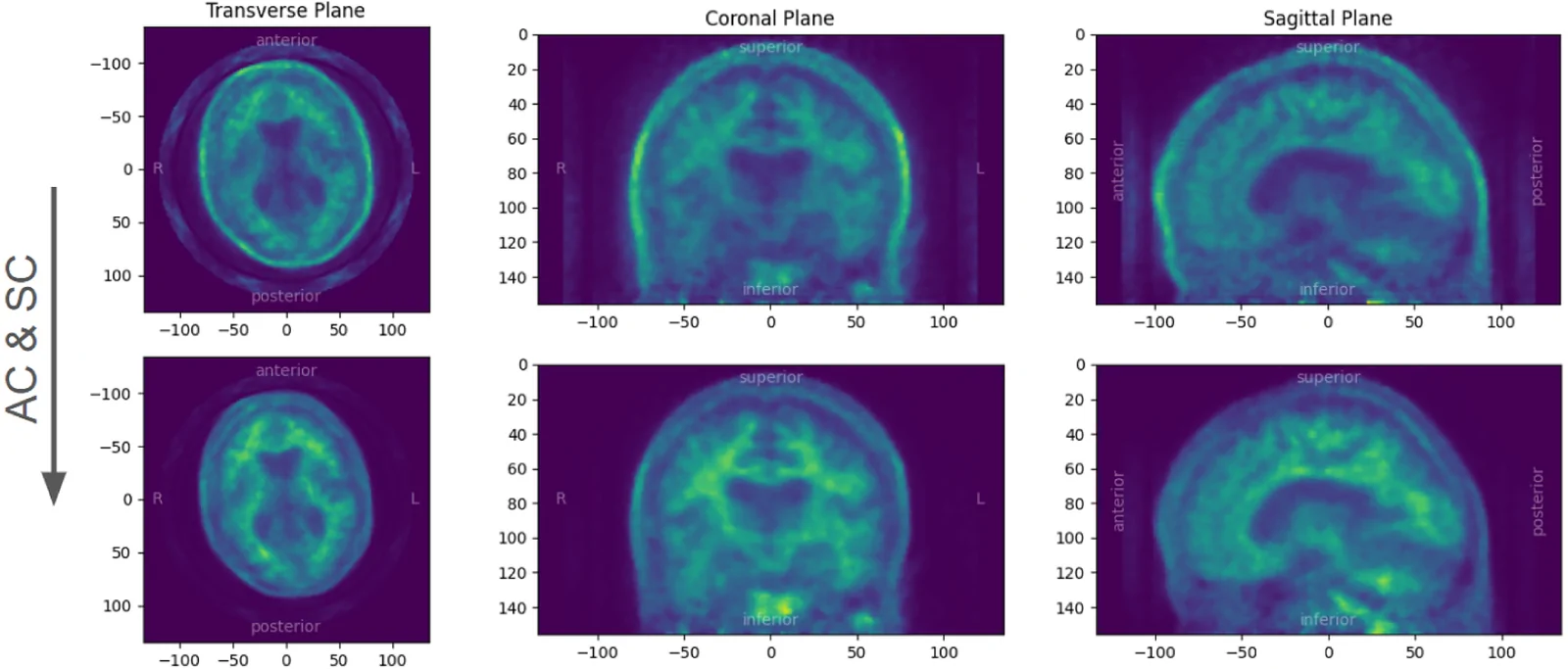
PET images of the same patient injected with ${}^{18}$F-florbetaben, reconstructed without AC and SC (top) and with CT-based AC and SC (bottom).

Attenuated photons from one LOR may still be detected in another LOR as a scattered coincidence. Therefore, scatter correction (SC) and attenuation correction in PET are inherently linked, and the two most common scatter correction methods, single scatter simulation [20] and Monte Carlo simulation [21], both take an attenuation map as an input to guide the algorithm. The fraction of measured scattered coincidences can be reduced by improving the energy resolution of a PET detector, but there are physical limitations to this approach, such as the statistical nature of the measured signal and inherent noise in the readout electronics [18]. The impact of performing AC and SC during PET reconstruction is visualised in Fig. 2.

## 3 Quantification of AC performance

The performance of AC methods in PET imaging is commonly assessed by comparing the generated attenuation maps or the attenuation corrected PET images against reference data. Given the widespread adoption of PET/CT scanners and straight-forward translation of the X-ray energy CT images to 511 keV μ-maps [12], [22], CT-based methods have replaced transmission scans as the reference standard [14], [17], [23].

### 3.1 Metrics for AC evaluation

Evaluation metrics can be broadly categorised into intensity-based metrics, which quantify differences in image intensity, and structural metrics, which assess perceptual or structural similarity between images. Additionally, regional metrics are more clinically relevant as they measure errors in diagnostic quantification.

1. **Intensity metrics** measure differences between the predicted and reference image/attenuation map on a per-voxel basis and provide quantitative estimates of the residual error. These metrics include:
  • Mean Square Error (MSE) or Root Mean Square Error (RMSE) which directly compare intensity differences.
  • Peak Signal-to-Noise Ratio (PSNR) that evaluates the ratio between the maximum possible signal intensity and the MSE, where a higher PSNR indicates a higher similarity with the reference image [24].
  • Correlation Coefficients that assess linear agreement between predicted and reference intensities. They are insensitive to global intensity biases and low correlation coefficients may indicate discrepancies in the spatial distribution of activity compared to the reference.
2. **Structural metrics** reflect perceptual similarity and overlap.
  • Structural Similarity Index Measure (SSIM) correlates better with human visual assessment [24], [25]. It takes into consideration luminance, contrast and structural similarity.
  • Dice Coefficient (DC), while being a voxel-wise metric, provides a measure of how well tissue boundaries align, especially when attenuation maps are segmented into tissue classes (air, soft tissue, bone). It measures the overall overlap between binary images, where a DC of 1 indicates perfect alignment. This metric can be sensitive to small regions, such as thin structures like the skull.
3. **Regional and clinical metrics** focus on PET signal quantification in anatomically defined volumes of interest (VOIs). These metrics are generally calculated on voxel intensities, SUV values, or SUV to reference values (SUVr).
  • Correlation analysis of SUV, SUVr, and derived tracer specific methods (such as Centiloid [26]) is particularly relevant in clinical studies for diagnosis and monitoring.
  • Bland–Altman Analysis [27], [28] provides agreement and bias assessments between PET quantification using computed and reference AC.
  • Percentage relative or absolute errors within different tissue classes (e.g. water-based soft tissue, fat-based soft tissue, bone, air) highlight tissue-specific biases in images.

All three categories are important for evaluating AC performance in PET imaging. General voxel-level intensity accuracy in the AC PET ensures reliable quantification of tracer uptake (global bias), while regional metrics ensure that the AC does not introduce spatially varying errors (local bias) that can affect quantitative readings.

### 3.2 Error propagation

The translation of voxel-level errors in attenuation coefficients to errors in the final AC PET image is non-trivial. Small errors in the attenuation map, such as those resulting from registration errors in template-based methods, segmentation or deep learning approaches introduce primarily local inaccuracies at the bone-air or bone-soft tissue boundaries in the AC PET. On the other hand, biases in attenuation coefficients and missing anatomical features (e.g. the skull or air cavities) can propagate to biases in larger parts of the AC PET through the line integrals used to compute the ACF.

Consequently, the choice of evaluation metrics should ideally be guided by the goal of the application. If the goal is to have qualitative PET images for visual reading, then structural similarity and voxel-wise errors are appropriate. For quantification, such as computing regional SUVr, volume of interest metrics may be more appropriate, since local voxel-wise inaccuracies may only have marginal effects on average intensity levels in brain regions [29].

## 4 Segmentation methods

**Fig. 3:**
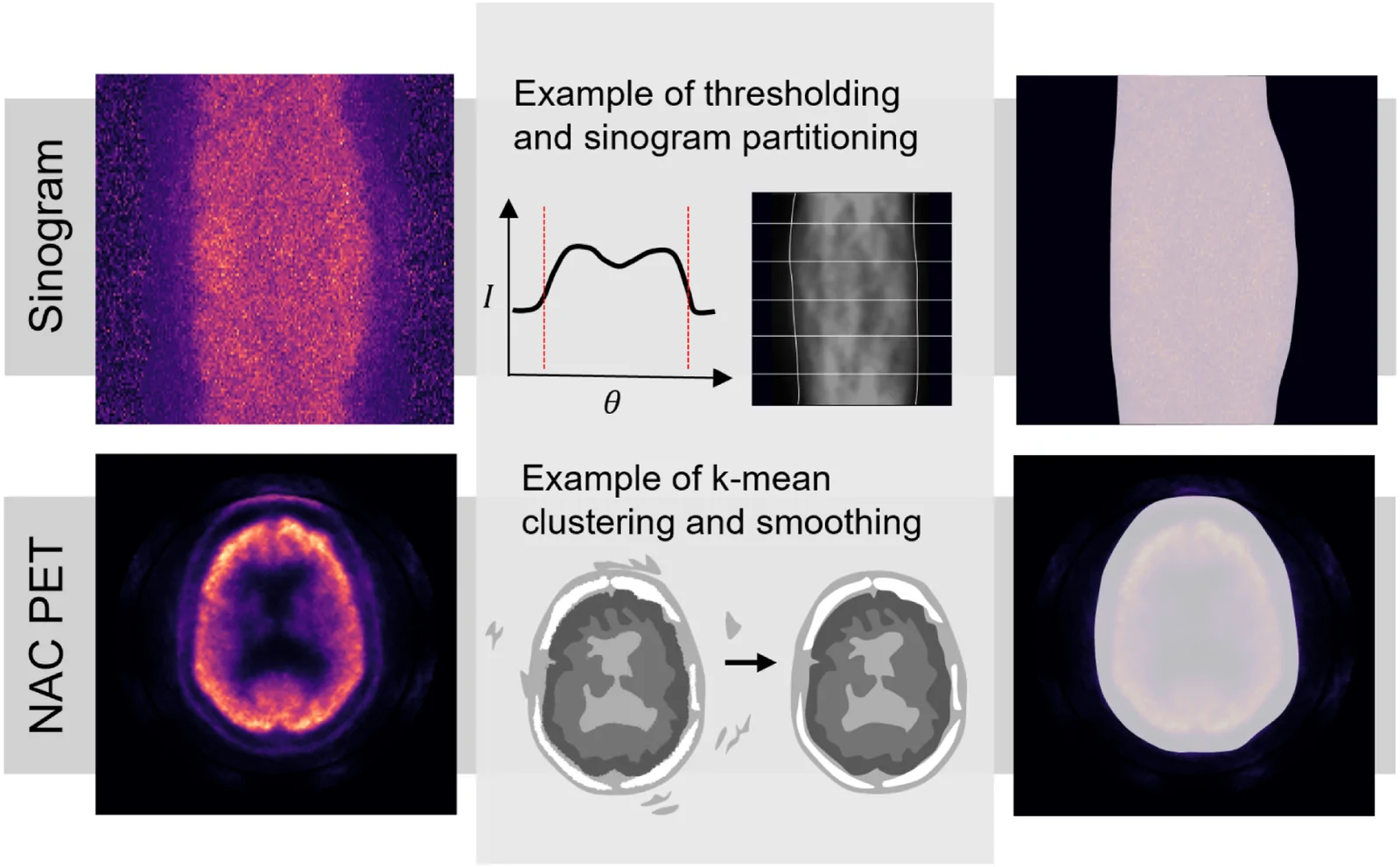
Illustration of segmentation-based methods for PET AC inspired by [30], [31], [32]. The uniform attenuation correction factors and attenuation maps obtained from the segmentation approach are then used for attenuation correction.

Segmentation-based methods for attenuation correction are attractive for their simplicity and computational efficiency. The general concept is to delineate the anatomy directly from the acquired data. This can be done on sinograms [30], [31], where no initial reconstruction is required, or on a preliminary non-attenuation corrected (NAC) PET image [6], [32] (Fig. 3). Common methods used in these approaches include thresholding, edge detection, morphological opening/closing, hole filling and connected-component analysis.

Early implementations worked directly in projection space, generating attenuation maps from emission sinograms without reconstruction, offering computational savings. [31] introduced one of the first fully automated approaches, combining sinogram-based contour detection with a polygonal contour tree to represent head, skull, and head holder. [30] later developed a robust edge-detection algorithm for head contouring in the projection domain, based on sinogram partitioning and local thresholds, assuming a fixed skull layer around soft tissue. Their method achieved agreement within 10% of transmission-based correction across tracers and uptake distributions. While simple and broadly applicable, both methods had limitations: [31] depended on extra-cranial uptake, while [30] simplified bone as a uniform layer, leading to residual bias.

More generally, sinogram-based segmentation is sensitive to tracer distribution and noise due to its lack of anatomical context. This is addressed by performing the segmentation on a preliminary NAC PET image, using the reconstructed activity distribution to delineate tissue boundaries. Soriano et al. [6] demonstrated this approach in dedicated breast PET, where the nearly homogeneous attenuation facilitated robust contour extraction. More recently, [32] compared various image quality metrics between their segmentation-based AC method on a brain-dedicated PET scanner and a PET/CT system, assessing both emission-based and CT-based attenuation correction. Their method applied k-mean clustering followed by post-processing to the NAC PET image in order to generate a smooth contour of the head. The comparison revealed that 11 out of 27 brain regions showed statistically significant differences in SUVr values when segmentation-based AC was used instead of CT-based AC.

Segmentation approaches are particularly well suited for applications where the imaged body region exhibits approximately homogeneous attenuation (e.g. breast [6]). On the other hand, assigning uniform attenuation coefficients to the head, or omitting the skull, has been shown to introduce bias in brain PET imaging [13]. This can be partially alleviated by applying a fixed skull thickness surrounding the segmented contour [30]. A primary limitation of segmentation methods is their intrinsic dependence on radiotracer distribution, which reflects metabolic function rather than true anatomical boundaries. In cases of atypical or pathological tracer uptake – such as in neurodegenerative diseases or brain tumours – algorithms based on intensity thresholding or edge detection can fail.

Segmentation methods are often used as a starting point for more complex methods, such as MLACF [33] and deep learning applications [19], [34], [35].

## 5 Template-based attenuation correction

Template- or atlas-based attenuation correction methods rely on external image data with corresponding attenuation maps, which are spatially registered to the data of the new patient under examination, so that the external attenuation information can be applied. The terms *template* and *atlas* are at times used interchangeably in literature, with a general tendency of atlas referring to more generic population-based models derived from multiple samples containing segmented tissues or anatomical regions of interest, and template referring to physical data such as attenuation values or CT images [13], [29], [36], [37]. Similarly, registration of image data is also often called *spatial normalisation*. While the former tends to refer more to aligning two images, the latter refers more to aligning multiple images to the same common reference space [38].

Atlas registration methods are very established for segmentation tasks [39] and were consequently also used with the advent of PET/MR scanners to translate the MR image to tissue labels and ultimately to attenuation maps [13], [14], [40]. For the purpose of generating only an attenuation map, the advantage of using templates directly is that no annotated atlas datasets need to be created and that weighting multiple templates is straight-forward [36]. Since atlas/template registration is quite a mature technique [41], novel research is nowadays primarily done on machine learning, be it in image registration (review: [42]) or in the more obvious directly learned attenuation map estimation (Section 7). Unfortunately for PET-only applications, most atlas/template registration techniques work on MR data, where the abundance of anatomical information facilitates the estimation of even non-linear deformable transformations.

### 5.1 Single-template registration

**Fig. 4:**
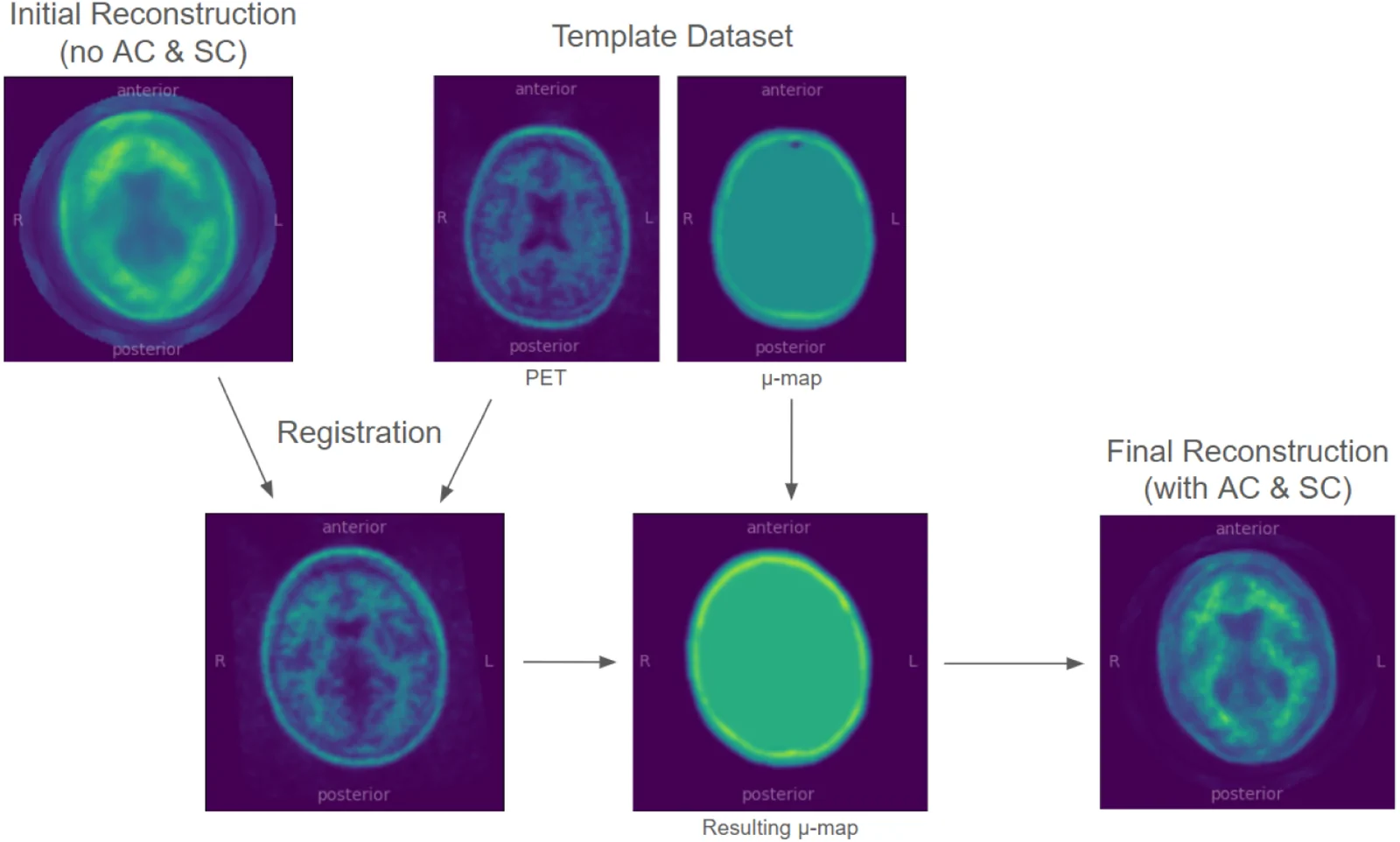
Illustration of the template-based correction workflow used in [5], employed using affine registration on a ${}^{18}$F-florbetaben amyloid PET scan.

If only PET data are available, non-linear registration algorithms commonly do not perform very well, due to the much scarcer anatomical information that is available in a PET image and due to tracer- and pathology-dependent differences in activity distribution [5], [43]. Consequently, PET-only atlas/template registration methods usually perform a linear affine transformation as a first step, which can be represented by a global matrix multiplication encoding rotations, translations, stretching and scaling [5], [43] (Fig. 4). Optionally, a constrained non-linear transformation is performed in a second step [43], [44], [45]. This additional non-linear update is often performed in PET image quantification applications, where it is paramount that brain regions are correctly identified. For the purpose of attenuation correction, the accuracy of brain region placement has a lesser impact and non-linear registrations may be less important [5], [29]. On ${}^{18}$F-FDG PET scans – which among the commonly used PET tracers contain the most amount of anatomical information –, a non-linear registration using SPM2 has been employed after an initial affine registration [46], [47]. The authors state that the PET images looked visually identical to transmission-based attenuation correction, but that certain brain regions showed statistically significant bias (< 7% relative errors on VOIs).

Different PET radiopharmaceuticals distribute very differently in the brain, requiring separate templates for each tracer. [48] developed a template for tau PET to enable a voxel-wise analysis of Alzheimer’s disease in a common frame of reference, i.e. the Montreal Neurological Institute (MNI) space. They show that the tau PET template performs comparably well to MR-based spatial normalisation. An extensive review of tracer-specific PET templates for image quantification was written by [49].

Apart from the tracer, certain pathologies can alter the anatomy of the head enough to warrant the need for a pathology-specific template. [50] developed a PET template for Down syndrome ${}^{11}$C-PIB images, because MR images were deemed structurally too different from existing PET/MR templates to be useful, and also because MR images were not always available. Similarly, it was found that for pediatric patients it is important to adjust template or deep learning based methods to the very different head anatomy compared to conventional head templates [51]. Also age and population differences can warrant the need for specific templates [49].

### 5.2 Multi-template registration

While the methods using just one template cited above showed that remaining inaccuracies of the attenuation map had at most a minor impact on the image quality – as assessed by VOI analysis of the PET images – using multiple templates is commonly expected to yield superior results because they can cover a wider range of geometries [49]. In the related field of biomedical image segmentation, [39] wrote an excellent review summarising the approaches for selecting the best atlas/template, or how to combine multiple into one segmentation. Many of these methods, while developed for MRI or CT, could in theory also be applied for attenuation correction in PET:

• All templates are registered to the novel image independently, and the resulting attenuation maps are averaged. The computational cost can be reduced by pre-registering the templates and concatenating these template-to-template transformations onto an initial novel image to template transform. These optimisations come at the cost of decreased registration quality.
• Preparatory offline learning of the templates can help identify more reliable templates that can be weighted higher (e.g. [52]), training a support vector machine on the templates to find the closest matches [53], identify regional characteristics that guide the choice of the most appropriate template per region [54], [55] and similar approaches.
• The characteristics of the deformation between the template and the novel image can be used in the selection of the most similar template (e.g. a distance metric of the transformation to an average image can be computed to look up the template that has the closest distance [56]).
• Pre- or post-registration, image similarity metrics can be used to find the best templates, e.g. sum of squared differences and cross-correlation have been shown to be reliable metrics for MRI [57], [58]. More complex metrics, such as principal component analysis [59] or clustering [60] have also achieved good results.

One difficulty in generalising the above-mentioned techniques to template PET registration is the variability in PET tracer uptake between patients and between tracers. Another difficulty is, that PET images generally contain much less anatomical information than CT or MR images, which means that local image comparisons can be unreliable.

We currently do not know of publications specifically investigating multi-template registration for attenuation correction in PET-only scanners. With PET data only, multi-template methods have primarily been used in the field of spatial normalisation for image quantification, where correct alignment of brain regions is more important than for attenuation correction. In this field, MR data are commonly used for identifying the brain regions, but due to PET/CT systems being more common there was an incentive to find a PET-only approach for spatial normalisation [48].

For β-amyloid (Aβ) PET tracers, the distribution varies considerably based on Alzheimer’s disease (AD) progression. While the tracer is primarily visible in white matter structures for AD-negative scans, AD-positive scans show strong tracer uptake in the grey matter [61], [62]. Quantification of AD progression is commonly done by computing the SUVr between AD-indicative regions and a reference region (commonly the whole cerebellum (WCB), the pons or cerebellar grey matter). The SUVr-based Centiloid scale [26], which aims to unify AD quantification across tracers and imaging protocols/devices, has become the de-facto standard for diagnosis and treatment monitoring [4], [63], [64].

While the standard approach for computing Centiloids performs MRI-based registration, there have been several PET-only methods that have shown comparable performance [43], [44], [45], [65]. They all follow a similar strategy, in creating a fully positive and a fully negative amyloid distribution, that can then be weighted to best match a novel image to create a template that is as close as possible to the observed distribution. [43] spatially normalised 72 ${}^{18}$F-flutemetamol PET images using corresponding MR images to MNI space and then performed a voxel-wise linear regression with respect to the AD-indicative composite SUVr scores. This gave them an image of the intercept and an image of the slope. Adding the slope image with a scaling factor between 0 (Aβ negative) and 1 (Aβ positive) gave them the ability to optimise the template within their initial affine registration. Afterwards, they perform another rigid registration of the reference region containing pons, WCB and structures in between, since the authors comment that a non-linear registration has too many degrees of freedom given the information content of PET images. This method was compared to SPM’s MR-based spatial normalisation and gave comparable results with even an improved registration in the cerebellum region. Furthermore, the ${}^{18}$F-flutemetamol template worked equally well on ${}^{11}$C-PIB PET data, indicating that different amyloid tracers do not necessarily require individual templates.

[66] used a very similar approach, where they computed an average Aβ positive and Aβ negative atlas and weighted them linearly to maximise the normalised mutual information between the atlas and the target. They compared this method against a ground-truth MR-based registration for data from five different amyloid tracers and additionally for ${}^{18}$F-FDG. For ${}^{18}$F-FDG, they directly performed a non-rigid registration to the atlas, without any weighting of different templates. They report mean absolute percentage errors for the quantification below 5% for all tracers. In a later study, the method was also shown to be accurate to within 2% for most amyloid tracers for computing Centiloid values [45]. This method was independently validated by [44] with a different computing environment and different datasets and showed good correlation to the MR-based standard approach, with very few registration-based outliers.

Following on from the above methods, [37] propose to generalise the multi-atlas approach to have a collection of probabilistic brain regions that are selected for each new image based on a gaussian mixture model. The advantage of this approach is that it is not specifically linked to one tracer and could also work well for a new unseen tracer. They report a better match to the “ground truth” MR-based registration than using a general template per tracer (obtained by averaging around 10 PET scans per tracer and pathology). In a VOI SUVr analysis they report 2.12% errors with their proposed method and 3.28% with the per-tracer templates.

One interesting recent approach to generate an attenuation and scatter corrected PET image from an uncorrected PET image that falls roughly into the field of multi-template attenuation correction was presented by [67]. They use a 3D rendering technique from computer vision to learn a joint representation of a corrected and uncorrected PET consisting of randomly initialised Gaussians. A novel uncorrected PET is then used to fine-tune this representation, resulting in a corrected PET. The results of the technique were not as good as for the U-Net or diffusion model (e.g. RMSE 0.0453 compared to 0.0334U-Net; PSNR 25.57 compared to 29.65), but it offered higher flexibility and reduced dependence on large training datasets. Unfortunately, the method was not compared to a conventional template-based AC.

### 5.3 Considerations for template-based attenuation correction

Template/atlas based attenuation correction methods for brain PET commonly report VOI-based biases below 4%–5% [29], [47], with some specific brain regions occasionally showing relative errors of up to 8% [47]. One obvious limitation of template-based methods is that they are not equipped to deal with uncommon geometries, such as missing parts of the skull, uncommon skull thickness or metal implants that significantly alter the attenuation distribution in the field of view [68], [69] (in these cases, patients have most likely undergone CT imaging after surgical interventions or for diagnostic purposes, and this CT can be used for AC instead). Even dense hair was shown to lead to significant quantification errors in the back of the head [70].

While template-based attenuation correction is very valuable in brain imaging due to the clearly defined, rigid anatomy, for the torso or other body parts it is much harder to implement, since distinct features like lungs, heart and bones are more loosely coupled to soft tissues like skin, fat and muscles.

### 5.4 Registration algorithms

An essential component of template registration methods is the actual image registration algorithm. The algorithms can generally be categorised by the cost function they seek to minimise/maximise, the nature of the image deformations they perform and by the deployed optimisation strategy.

The most relevant characteristic of registration algorithms in the context of attenuation correction is the transformation model. Rigid registrations are commonly done in head attenuation correction when there is a direct link between two images, e.g. when registering a pre-acquired CT image to a newly acquired PET image of the same patient. Affine transformations additionally allow for stretching and scaling, and are useful when registering a template to a new PET image of a different patient. Rigid and affine transformations are globally defined across the whole image, and are contrasted by deformable transformations, which define locally varying deformations to adjust for more localised differences in geometry between patients and templates. The increased degrees of freedom for deformable registration come at the cost of running a higher risk of overfitting, when the information content in the images being registered is insufficient.

Deep learning has been used to optimise all of the components of image registration mentioned above. The most straightforward implementation consists of learning a mapping from the moving to the fixed image to drastically increase registration speed. A further advantage of deep learning-based approaches lies in their ability to learn latent feature representations that capture high-level structural information (such as anatomical boundaries, tissue-specific appearance patterns and geometric relationships). When used to guide the prediction of deformation fields, these features help produce transformations that are more anatomically plausible and more robust to noise, an important aspect in PET attenuation correction. [40] give a very good overview of registration algorithms.

A wide range of established software packages for medical image registration are available. *SPM* [71], [72] (Statistical Parametric Mapping), provides Matlab-based implementations of linear and deformable image registration. C++ libraries such as *NiftyReg* [73], [74], [75], [76], *ANTs* (Advanced Normalisation Tools), and *elastix* [77], offer rigid, affine and non-linear registration capabilities, as well as GPU-accelerated workflows (NiftyReg) and bindings to other programming languages, such as *ITKElastics* [78] and *SimpleITK* [79].

## 6 Simultaneous reconstruction of emission and attenuation

Based on the observation that attenuated PET data inherently contain information about the distribution of attenuating tissues in the field of view, methods have been developed to extract attenuation and activity maps simultaneously from the uncorrected data. A detailed review of these methods was written by [80]. The two most commonly used algorithms are the maximum likelihood reconstruction of attenuation and activity (MLAA) and maximum likelihood attenuation correction factors (MLACF).

One of the most influential early approaches was the post- reconstruction correction method known as Chang’s method [81]. The core concept involved creating a pre-calculated, spatially-varying correction factor matrix, assuming a known body contour and a uniform attenuation coefficient within it. The uncorrected image was then multiplied by this matrix to compensate for attenuation. Chang’s method also included an optional second iterative step to refine the result by correcting and adding back a reconstructed “error” image. This technique critically relied on having an a priori definition of the patient’s attenuating boundary, and the assumption of a uniform attenuation coefficient.

### Maximum likelihood reconstruction of attenuation and activity.

**Fig. 5:**
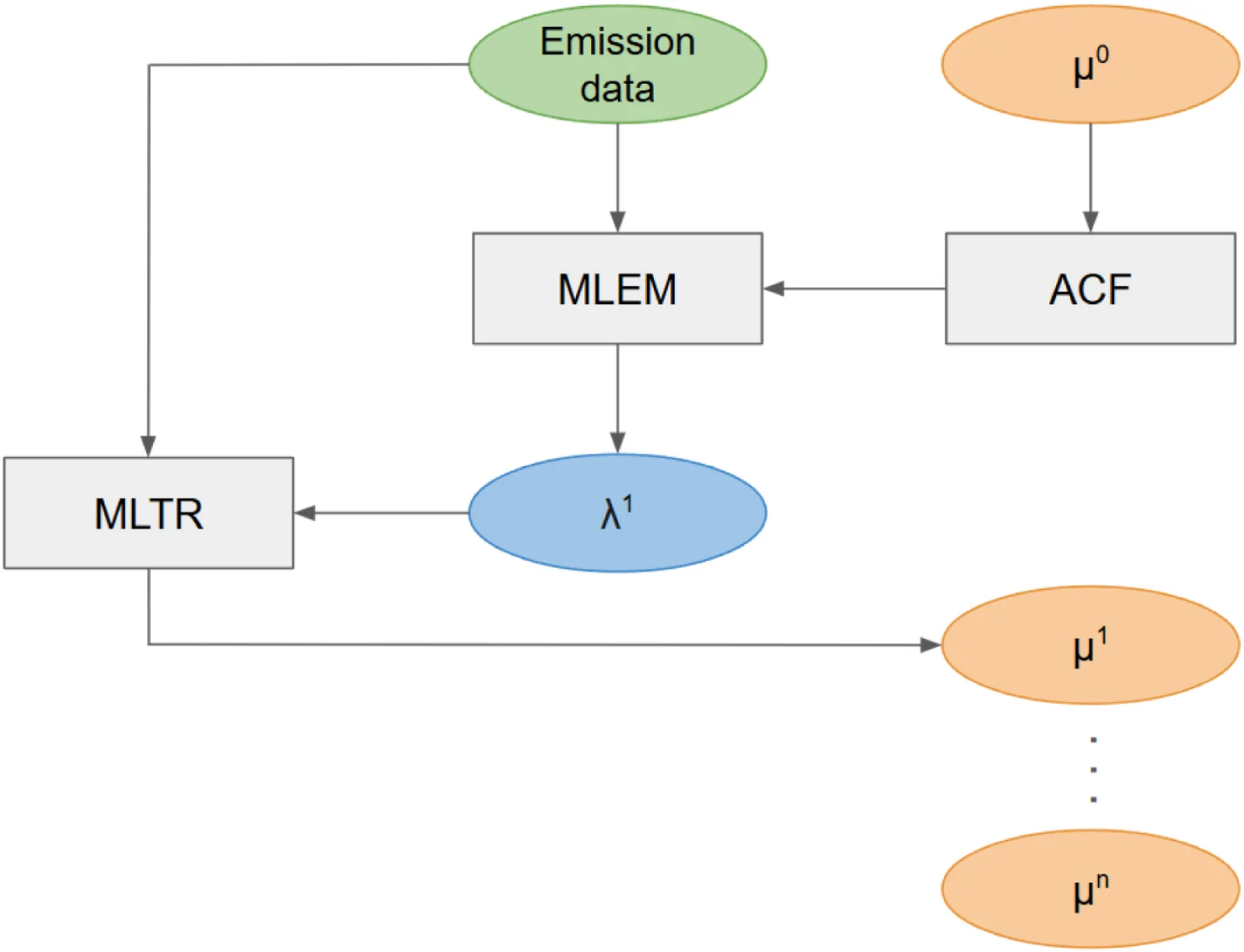
A diagram illustrating the structure of one iteration of the MLAA algorithm (as per [82]). The λ-circles signify the different iterations of the estimated emission, while the μ-circles are the iterations of the estimated attenuation map. Maximum likelihood expectation maximisation (MLEM) and maximum likelihood for transmission tomography (MLTR) are algorithms for updating emission and attenuation maps, but other options exist [16].

This method originally proposed by [16] alternates between updating the attenuation and the activity map (Fig. 5). Because of the interdependence of the two distributions, this iterative optimisation problem is non-convex and the algorithm can converge to a non-optimal solution, resulting in cross-talk between attenuation and emission in the result. It was found, that using time-of-flight (TOF) data can minimise this cross-talk significantly [83], which is why MLAA is nowadays primarily used on TOF scanners. One added difficulty of MLAA is, that it is theoretically only defined up to a constant [84]. There are various approaches to tackling the constant offset issue using anatomical assumptions (water) or data (atlas, MR) [14], and most report errors below 7% in the brain. [85] reduced the degrees of freedom of MLAA by defining connected regions and only finding the attenuation coefficients for these. These regions were segmented from the patient MRI and initialised to the attenuation of water, but potentially the regions could also be derived from a registered template. Importantly, MLAA does not include scatter or randoms correction, and these need to be approximated before reconstructing the attenuation and emission maps. Inaccurate scatter correction or physical modelling of the scanner can introduce cross-talk even when using TOF PET data [86].

### Maximum likelihood attenuation correction factors.

Instead of reconstructing attenuation maps, the MLACF algorithm directly aims to optimise the attenuation in projection space, which makes it faster compared to MLAA [87]. This method has already been evaluated for CT-less brain imaging on conventional PET/CT systems [88], [89], showing that as few as 2–3 TOF bins can be sufficient for the estimation of the attenuation correction factors — an important finding for brain imaging given the head’s smaller size compared to the whole body. On the other hand, [88] observed artifacts the attenuation maps at the boundaries of the active areas. Another drawback is, that data consistency is not guaranteed, which becomes more pronounced for long axial field of view scanners, where the number of LORs grows quadratically while the number of voxels in the attenuation map grows linearly [80].

Recently, MLACF was used as one part of the image reconstruction pipeline in a commercial brain PET scanner, where the MLACF attenuation correction factors were reconstructed and the brain region obtained from thresholding of an initial activity image was scaled to align the attenuation map histogram with the attenuation factor of soft tissue [33].

An important consideration for the use of MLAA and MLACF is that the methods become less stable in regions with low activity concentrations [80], [89]. This could impact the performance of these methods for PET tracers with very localised uptake characteristics, such as tau.

## 7 Deep learning

**Fig. 6:**
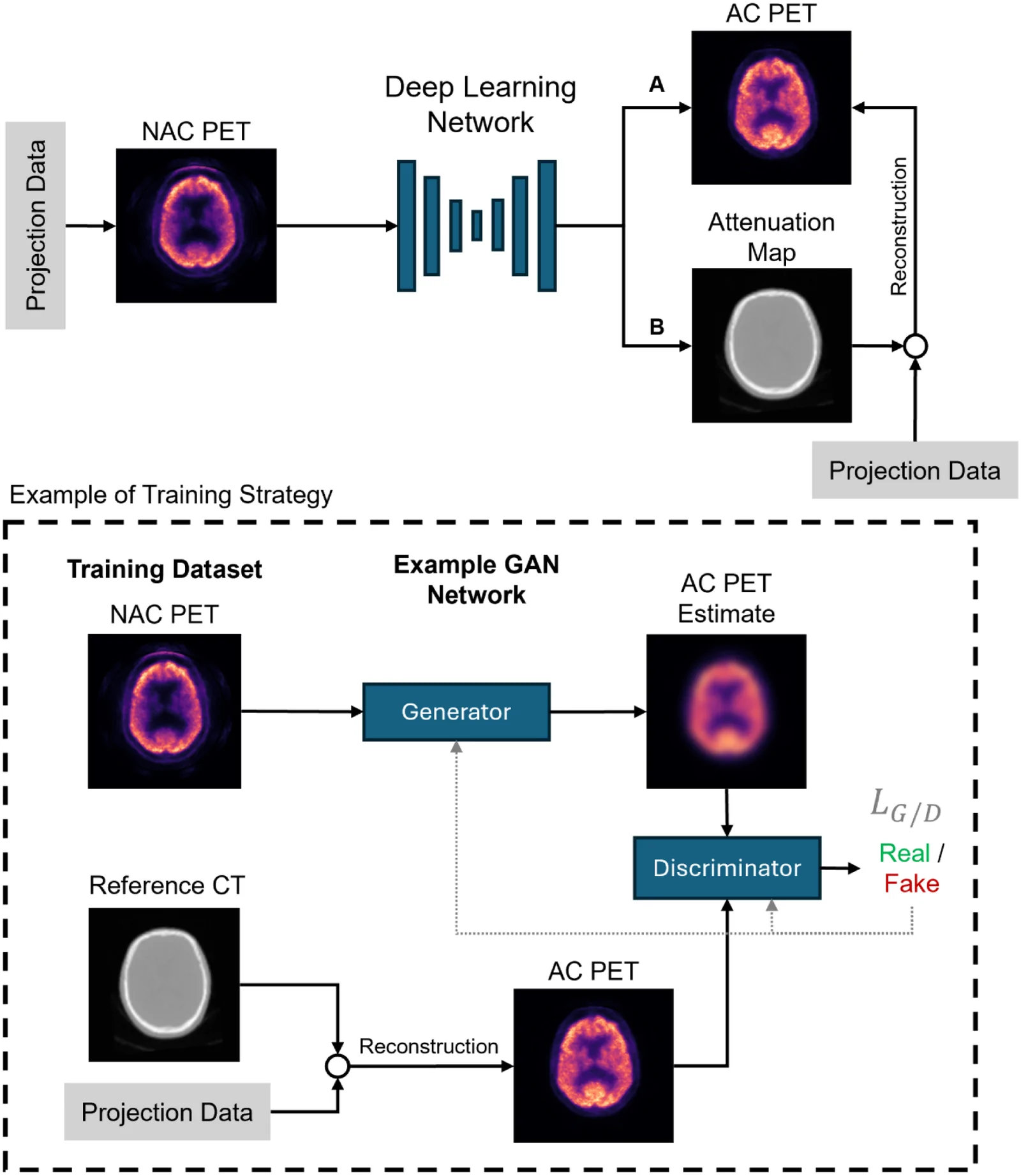
Illustration of two deep learning-based approaches for attenuation correction in PET imaging. Path **A** depicts the direct prediction of AC PET from NAC PET inputs. Path **B** represents the indirect approach, where synthetic CT-like attenuation maps are first generated and subsequently used in conventional reconstruction algorithms for attenuation correction. The lower panel presents an example training strategy using a GAN, where the generator predicts AC PET from NAC PET inputs, and the discriminator is trained to distinguish between the generated images and the ground truth AC PET images derived from reference CT-based attenuation correction.

With the advances in machine learning, data-driven approaches for AC using deep learning have been extensively investigated, particularly leveraging PET/CT data. These methods learn mappings from an input distribution (e.g. non attenuation corrected (NAC) PET) to a target distribution (e.g. synthetic CT) by optimising a chosen objective function. Although these approaches require additional paired CT data during training, they remain applicable in practice to PET-only scanners (Fig. 6).

The two most common problem formulations are:

1. **Prediction of synthetic CT** from NAC PET images or MLAA μ-maps, where the synthetic CT is then used to derive an attenuation map for AC (path **B** in Fig. 6).
2. **Prediction of AC PET**, where the attenuation map is bypassed and the AC PET is directly predicted from NAC PET (path **A** in Fig. 6).

A previous review comprehensively summarised deep learning AC methods published up to 2022 [17]. In this section, we highlight emerging trends and recent advances in the field, focusing specifically on approaches that directly address PET attenuation correction, while excluding methods aimed solely at generating attenuation maps from MR data.

### 7.1 Deep learning networks

The most widely used deep learning network architectures for PET AC are convolutional neural networks (CNNs), particularly U-Net-based encoder–decoders [90], and generative adversarial networks (GANs) [91]. U-Net and its variants encode the input (e.g. NAC PET, MLAA μ-map) into a latent representation and then decode it to generate an output image (e.g. synthetic CT or AC PET), that is then compared to a ground truth Ref. [35], [92], [93], [94], [95], [96], [97]. Skip connections linking the encoder and decoder preserve finer details. GANs extend this framework by introducing a discriminator network that learns to distinguish synthetic outputs from real ground truth (e.g synthetic CTs vs ground truth CTs) [98], [99]. This adversarial training encourages the generator to produce images with improved realism and sharper anatomical boundaries compared to CNNs alone. More advanced models have also been proposed, such as CycleGANs [100] and Invertible Networks [101] that learn the forward and backwards mappings to enhance generalisability and robustness. Although CycleGANs are more complex to implement, they are particularly advantageous in PET attenuation correction, because they do not require perfectly aligned PET/CT data. The cycle consistency constraint enables training with unpaired or imperfectly aligned datasets, allowing the model to tolerate minor motion-induced PET/CT mismatch and reducing the risk of learning anatomically incorrect correspondences. Beyond the network architecture, another relevant design parameter is the choice of objective function, ranging from simple voxel-wise losses to hybrid approaches combining structural similarity, perceptual losses or task-specific metrics. The training strategy (e.g. paired vs. unpaired learning) and dataset size and quality are also very relevant.

The growing interest of the deep learning community in cross-modality image synthesis (e.g. PET-to-CT) has accelerated the development of advanced architectures [102], [103]. While we discuss the networks that were designed to produce realistic CT images from PET data, we focus on approaches that address attenuation correction in PET, emphasising design choices and evaluation strategies that directly impact clinical translation. Although our primary focus is brain PET, we include methods trained and tested on full-body PET that are transferable to brain PET.

### 7.2 From NAC PET to synthetic CT

Under this configuration, the network is trained to output a synthetic CT from NAC PET or MLAA outputs.

#### Towards more realistic CT images.

Instead of using the information from the entire NAC PET image, [102] decomposed the synthesis problem into structural and texture components through a dual stage generator with respective structure and texture loss functions. In addition, a local discriminator is used to ensure feature consistency. The network (DSG-GAN) was trained and evaluated on brain PET/CT images from 112 patients, producing very high quality synthetic CT scans, achieving a PSNR and SSIM of 29.61 and 0.899, respectively. The authors provide a very comprehensive ablation study, detailing the added benefits of having a dual-stage generator, deep-perceptual connections, and a local, context-aware discriminator. Their method delivers higher performance than numerous other CNN and GAN based architectures, but it is unclear if the added detail on the generated CTs has a practical benefit for PET AC, which generally has a lower resolution.

Similarly [101], focuses on improving texture and structure by presenting an Invertible Neural Network architecture that learns the bijective mapping between NAC PET and CT. The network was trained on brain PET images from 37 patients, and tested on 2D slices from 5 patients (200 slices). The authors report a PSNR and SSIM on the synthetic CT scans of 24.00±1.53 and 0.812±0.040, respectively. In addition, they compare the AC PET image using their predicted attenuation map against the one obtained with the reference CT and report a MAE and RMSE of 0.0241±0.0047 and 0.0641±0.0116 respectively, surpassing the results using CycleGAN and Pix2Pix.

#### MLAA as an input.

As an alternative to obtaining NAC PET images through OSEM reconstruction, other methods have proposed to use MLAA (Section 6) attenuation maps (μ-MLAA) and PET activity (λ-MLAA) as input to the networks. These methods have primarily been evaluated in full body PET, and require time-of-flight information.

For instance, [96] use a modified version of the 3D U-Net and in addition to an image domain loss and an image gradient difference loss, they include a projection domain line-integral loss that works at a patch level, where the predicted attenuation coefficients are compared with the CT derived attenuation coefficients in full body PET/CT. They report a PSNR on the attenuation map of 30.35±1.53 and a normalised RMSE and relative error of 0.0647±0.01 and -1.04±0.76%. Their ablation study shows that projection domain evaluation metrics of the attenuation map are better indicators of PET quantification accuracy.

Similarly, an interesting approach is proposed by [103], where they train a network (POUR-Net) to output CT-based attenuation maps from μ-MLAA and λ-MLAA and then refine the estimated attenuation map with a CT-derived attenuation map from an independent population of 2000 CT images. The closest resembling CT-derived attenuation map to the interim attenuation map prediction is non-rigidly registered to the μ-MLAA and λ-MLAA network inputs, and used as an input for subsequent training cascades in a multi-step training process. The refinement of the attenuation map with the external CT database increases PSNR of the attenuation map from 34.13 dB to 37.21 dB, where the reported U-Net approach has PSNR of 33.75.

When compared with the ground truth attenuation correction obtained from the CT, their proposed method translates to an increase in PSNR in the estimated AC PET image from 43.69±2.11 to 44.88±2.23 (U-Net 43.38±2.10). Although this approach has been evaluated only in full body PET, it would be interesting to quantify the advantages of using an external unpaired brain CT database for attention map refinement.

#### 2D or 3D networks.

Training networks on 2D axial brain images is generally more computationally efficient and requires less time than training 3D networks. However, at inference, 2D networks can create discontinuities in the axial direction, which are more significant when directly estimating AC PET images. [104] compare the performance of three different U-Net configurations (2D U-Net, 2.5D U-Net and 3D U-Net) in the task of CT synthesis from λ-MLAA and μ-MLAA brain ${}^{18}$F-florbetaben Aβ images. They perform 5-fold cross validation on 100 PET/CT samples, using 20 samples for testing on each iteration. The 2.5D U-Net configuration took as an input neighbouring transaxial slices to predict one 2D CT slice. Although the neighbouring slices were intended to minimise discontinuities, they did not improve performance over the standard 2D U-Net configuration. On the other hand, the patch based 3D U-Net took 6 times longer to train but showed increased performance when compared to the 2D U-Net, in both the synthesis of the CT (about 15% increase in bone DC and more than 50% increase in air DC) and on the AC PET calculated from the synthetic CT (PSNR 38.77 dB vs 35.73 dB).

### 7.3 From NAC PET to AC PET

Instead of predicting attenuation maps from NAC PET, this step can be skipped altogether and deep learning networks can be trained to directly predict the AC PET images. The advantage of direct AC PET estimation from NAC PET images is that it can do joint attenuation and scatter correction, removing the need for additional reconstructions. If not stated otherwise, the performance evaluation metrics (e.g. PSNR and SSIM) are calculated with respect to the AC PET obtained with the corresponding CT.

One research group first presented Deep-DAC, an encoder–decoder architecture with skip connections, to estimate brain AC PET slices from NAC PET slices with a simple L2 loss function [93]. The model was evaluated on brain PET images of 18 patients and showed a PSNR and SSIM of 39.22±3.65 and 0.989±0.006 respectively.

Similarly, [105] trained a modified version of a CycleGAN on full body NAC PET images from 20 patients and tested on 102 other patients. On the head and neck, they report a PSNR of 36.90±5.44 and a SSIM of 0.980±0.036. Their main modification was to include anatomical priors into the discriminator of the network and a corresponding body site loss which improved PSNR and SSIM from 33.5 dB and 0.910 to 37.5 dB and 0.970, respectively. This surpassed previous methods that used pix2pix networks trained on full body PET [106].

Another group evaluated the benefit of performing a simple segmentation-based AC on the PET images before using them as an input to a HighResNet to predict AC PET images [34]. The two class attenuation map used to pre-correct the PET image was obtained from the NAC PET image. The different input modalities were evaluated through a 4-fold cross-validation in 80 brain PET/CT images. Performing the preliminary attenuation correction on the PET image increased PSNR from 19.08±3.18 to 20.98±3.68, while the best result was obtained when they combined as an input the NAC PET image and the pre-corrected PET image (PSNR of 21.48±5.35).

The same group had also previously investigated deep learning methods for attenuation correction in the sinogram domain [107]. They investigated a wide variety of configurations for TOF and non-TOF PET emission data, including the estimation of attenuation correction factors from NAC sinogram or directly the estimation of AC sinograms. They also investigated the effect of performing scatter correction prior to using the PET data as an input [95]. The evaluation was performed on brain PET images from 20 patients. Under all configurations, the attenuation correction strategies considering TOF information significantly outperformed non-TOF methods in all brain regions. For non-TOF PET, predicting the sinogram directly led to a lower relative error, however, the results were not significant for all metrics and brain tissues. Performing scatter correction as a pre-processing step did not result in more accurate attenuation correction.

### 7.4 Other approaches

The methods listed above generally require PET/CT datasets to train the network. Instead, [35] explores the feasibility of a method applicable for PET-only scanners that does not use PET/CT data for training. They train a modified U-Net with simulated NAC PET data built from the BrainWeb open source dataset to output soft segmentations depicting the linear combination of 4 different tissue components (background, cavity, soft tissue and bone). The output of the network was then used as an input for the scatter and random estimation and as an initial point for computing estimated attenuation correction factors using MLACF. The general structure of the brain was estimated correctly but the deep learning network missed anatomical details. Although the approach was only trained on 12 synthetic samples and tested on 3 real PET/CT scans, the relative error in the AC PET images compared to the standard CT-based AC PET images was below 5% for most brain regions, but exceeded 10% for bone regions and the sinus cavities.

### 7.5 Generalisability

A central issue in attenuation correction methods based on deep learning is the ability of the models to generalise across different tracers and clinical scenarios. Networks trained on a single tracer distribution may overfit to tracer-specific uptake patterns and perform poorly when applied to other tracers with distinct distributions.

To address this, [94] trained a CNN model on a large mixed dataset of six brain radiotracers and evaluated the performance not only on separate test sets of these six radiotracers, but also on ${}^{11}$C-Methionine, which was excluded in the training. The networks took as an input NAC PET images reconstructed using filtered back-projection and instead of using CT as a reference, the model was trained to estimate transmission scans obtained from a rod source. They compared the results of the model trained on this heterogeneous dataset against models trained per tracer. The study showed that training on a heterogeneous tracer dataset improved robustness: their estimated transmission scans had a mean PSNR of 29.15 dB across all radiotracer test sets, compared to 27.73 dB averaged over tracer specific estimates. As expected, the radiotracer-specific model trained with the largest data size more closely resembled the performance achieved with the model trained on a mixed dataset.

Similarly, [99] provides one of the most comprehensive evaluations of this issue using full-body PET/CT datasets acquired with different tracers and scanner vendors. Their results show that in the worst case, normalised RMSE increased by a factor of 6.1 and PSNR dropped by 13 dB when both tracer and vendor differ between training and testing. Their proposed approach – employing strategies aimed at improving robustness – outperformed other 2D and 3D deep learning approaches in out-of-distributions generalisation, demonstrating how their training design choices can help fight generalisability concerns.

As opposed to directly evaluating generalisability in predicted full-body AC PET images, [97] evaluated cross-tracer generalisability on predictions of CT-like attenuation maps from MLAA reconstructions and attenuation maps. Their findings highlight the importance of both dataset size and diversity, as reducing the training dataset by approximately 88% led to a six-fold drop in domain generalisability, while tracer-diverse training improved performance on completely unseen tracers, even when the dataset size remained fixed.

To the best of our knowledge, there are no PET-only papers investigating the effect of dataset size on the performance of deep learning algorithms trained for PET attenuation correction. However, [108] directly investigated the effect of deep learning training group size on attenuation correction accuracy in brain MRI-derived attenuation correction. The dataset consisted of 1037 adult PET/MRI scans with reference CT images. A modified version of the 3D U-Net was trained to output CT images from MRI scans. The effect of training group size was investigated by training 4 independent networks with increasing data (from 10 patients to 403). As expected, attenuation correction accuracy increased with training group size, where small group sizes led to smooth attenuation maps with a larger number of outliers. Surprisingly, the relative error across all brain regions quickly decreased from around -7% to below -4% as the training data increased to 100 samples.

### 7.6 General considerations

Deep learning-based attenuation correction methods have demonstrated strong potential for brain PET imaging, generally matching or outperforming other approaches. Reported voxel-wise errors are typically below 5% across brain regions, highlighting the ability of these models to provide clinically useful reconstructions. While direct comparison with template-based strategies remain limited, the current evidence suggests that deep learning achieves more spatially consistent estimates.

The choice of network architecture influences both the visual characteristics and quantitative accuracy of the generated attenuation maps. Simpler models such as U-Net or ResNet are relatively easy to train and generally produce robust outputs; however, they tend to yield smooth predictions that can exhibit global bias and may fail to capture fine anatomical details or subtle abnormalities, such as skull defects. By contrast, more complex architectures, like CycleGAN, are more challenging to train but can produce texturised outputs that recover local structure more faithfully when sufficient training data are available, albeit sometimes at the cost of introducing noise-like artifacts or hallucinations. Whether such texture artifacts affect attenuation correction performance significantly remains uncertain and needs further investigation.

Comparisons across published studies are complicated by differences in training and validation datasets. Factors such as the quality of the reference CT (registration errors or motion artifacts), dataset size, voxel resolution, acquisition timing and radiotracer type all contribute to variability in the reported results. Progress in the field would benefit from the establishment of a standardised, multi-centre PET/CT database specifically designed for brain imaging. Ideally, this would also include a dataset where the reference CT was acquired separately from the dedicated brain PET image, enabling more realistic evaluation scenarios for deep learning based attenuation correction in brain dedicated PET scanners.

Several methodological considerations also remain open. While some groups propose directly predicting AC PET instead of an intermediate attenuation map, this strategy relies entirely on deep learning and may be more sensitive to error, such as hallucinated structures from generative networks. Moreover, any improvements in the underlying reconstruction methodology would require retraining the network, since most NAC PET to AC PET deep learning models implicitly depend on the reconstruction pipeline during training. These factors must be weighted against the potential benefits of faster inference (e.g. <30 seconds for 3D encoder–decoder networks [109]) compared with conventional reconstruction pipelines, which would be relevant for clinical integration. From a clinical standpoint, predicting an attenuation map or CT image offers the advantage of providing an intermediate output that can be inspected for artifacts or inconsistencies, and it may also aid anatomical localisation during clinical readings.

Beyond methodological aspects, key limitations relate to generalisability. Most models are trained and tested on PET/CT acquired on the same machine, whereas deployment in brain-dedicated PET devices inherently involves out-of-distribution data. Furthermore, current training datasets often exhibit demographic imbalances, with under-representation of female participants, raising concern about potential bias in learned models [110].

## 8 Approaches used in commercial systems

CareMiBrain by MindView/OncoVision is a brain PET system supporting seated patient positioning. The detector is composed of monolithic LYSO crystals offering good depth of interaction resolution and has a field of view of 150 mm axially and 220 mm in diameter. The attenuation method used in the CareMiBrain scanner is similar to one published for breast PET imaging [6]. It is essentially an image segmentation method that employs denoising on a reconstruction without attenuation or scatter correction, followed by thresholding and postprocessing. Then, the identified patient volume is assigned the attenuation coefficient of water. The method was validated with 80 ${}^{18}$F-FDG patient scans by comparing VOI SUVr (relative to the whole cerebellum VOI) to reference PET/CT images, showing relative errors within 7% of the PET/CT Ref. [32].

NeuroLF by Positrigo is a dedicated brain PET system with an axial field of view of 163 mm and a diameter of 260 mm and LYSO crystal detectors employing a 4 by 4 light-sharing. The design emphasises an intuitive operation and a fast seated patient preparation. The attenuation correction in NeuroLF is computed with a template-based method using tracer-specific templates similar to the method described in [5]. The performance of the method was assessed using VOI comparison against reference PET/CT images for ${}^{18}$F-FDG patients imaged with a proof-of-concept system [8] with lower spatial and temporal resolution and a smaller axial field of view than the commercial NeuroLF device [5]. It showed comparable linear regression and correlation values when using different template datasets.

The PositView (SET-5002) system by Shimadzu Corporation [9] is a head and breast TOF-PET system with an axial field of view of 163 mm and a diameter of 300 mm [33]. The device uses MLACF to derive the attenuation correction factors from the PET data, combined with an offset attenuation map to scale the obtained attenuation map to the attenuation of soft tissue in the brain region [33]. Initially, the brain region was found by intensity thresholding of a NAC reconstruction, but other methods have been investigated [35]. In the commercial product, an improved version of the one presented in [33] is used. A prototype of the attenuation correction method was validated on patient data by measuring the variability in the attenuation maps obtained from PET scans with either ${}^{18}$F-FDG or ${}^{18}$F-flutemetamol (β-amyloid), showing that the relative errors in dementia-related VOI in the PET image were within 7% [111]. The largest differences in μ-maps were unsurprisingly observed in the skull.

The brain-dedicated VRAIN TOF-PET scanner by ATOX/QST employs a hemispherical detector arrangement with a maximum axial field of view of 224 mm and a diameter of 279 mm [10]. For the attenuation correction, VRAIN currently relies on pre-acquired CT images that are automatically modified to remove the headrest [112].

The PHAROS PET scanner by Brightonix Imaging is a multi-purpose system for scanning brains, breasts and extremities. It currently uses template-based and contour-based (calculated) methods for the AC.

In dedicated brain PET systems, the only additional object in the field of view is typically the headrest. Correction for the attenuation caused by the headrest has been studied e.g. by [112] and was not deemed essential. This is, however, dependent on the material and design of the headrest, and other systems model the headrest in the μ-map [33].

### 8.1 Considerations for commercial implementation

As this review highlights, there is a noticeable gap between the cutting-edge methods explored in academic research – particularly in deep learning – and the techniques currently implemented in commercial systems. While some of this delay can be due to the validation and regulatory timeline, it also reflects a fundamental difference in priorities between academic research and commercial product development. Understanding these priorities is key to bridging this gap.

An academic paper might highlight a method that achieves state-of-the-art average performance on a curated dataset. However, a commercial system must perform reliably on every scan. A method that is very accurate on average, but fails unexplainably in some edge cases, is clinically unviable. A simpler method that is consistently slightly less accurate, with predictable, detectable and bounded errors, is far more valuable. Furthermore, an attenuation correction method must be fully automated, computationally fast and seamlessly integrated in the reconstruction pipeline.

Deep learning methods show a lot of promise, but they also require large, diverse and meticulously curated datasets for training and validation. For a company, acquiring such multi-tracer data is a significant logistical and financial undertaking. In contrast, a template-based approach requires only the creation and validation of a few high-quality templates.

Academic researchers aiming for clinical translation can significantly increase the impact of their work by incorporating commercial-style validation. A thorough failure mode analysis – identifying and describing exactly when and why a method breaks down – is often more insightful for a potential implementer than a minor improvement in average RMSE or PSNR. By rigorously testing for robustness and clearly defining an algorithm’s operational boundaries, academic research can more effectively pave the way for true clinical innovation.

## 9 Conclusion

The development of dedicated brain PET systems has renewed the focus on attenuation correction (AC) methods that can operate without anatomical data from CT or MR. This review has surveyed the landscape of these techniques, from foundational segmentation and template-based methods to more advanced simultaneous reconstruction and deep learning approaches. Our analysis shows a clear evolution towards more patient-specific and accurate solutions, with each class of methods presenting a unique balance of strengths and weaknesses.

Template-based methods remain a pragmatic and widely used solution, offering robustness and computational simplicity that is effective for a majority of clinical scenarios with normal or near-normal anatomy. However, their reliance on a standardised model limits their accuracy in the presence of major anatomical abnormalities. Simultaneous reconstruction techniques like MLAA and MLACF leverage the emission data itself to derive an attenuation map, but their performance is sensitive to tracer distribution and noise, and they benefit significantly from good time-of-flight information, which is not universally available.

Deep learning has emerged as the most promising frontier, demonstrating the potential to generate highly accurate, patient-specific attenuation maps that rival those derived from CT. These data-driven models can learn complex relationships between emission data and tissue attenuation, offering superior flexibility. This power, however, comes with significant challenges, including the need for large, curated, and diverse training datasets, the risk of poor generalisation to out-of-distribution data (e.g., unseen tracers, rare pathologies, or different scanners), and the “black box” nature that can complicate clinical validation. The presence of foreign objects, such as medical implants or patient positioning devices, also remains a significant challenge for all non-anatomical methods.

Notably, a gap persists between academic research and commercial implementation. Current brain-dedicated PET scanners predominantly rely on established segmentation, template, or MLACF-based methods rather than the latest deep learning models. This reflects practical concerns regarding validation, regulatory approval, and the need for proven robustness in routine clinical workflows. Looking forward, the path to superior AC in stand-alone PET will likely involve hybrid approaches, such as using templates to initialise or constrain deep learning models. The development of standardised, multi-centre datasets and robust validation frameworks will be critical to building clinical trust and unlocking the full potential of data-driven AC, ultimately ensuring the quantitative accuracy and diagnostic value of dedicated brain PET imaging.

## CRediT authorship contribution statement

**Markus Jehl:** Writing – review & editing, Writing – original draft, Visualization, Validation, Supervision, Software, Resources, Project administration, Methodology, Investigation, Formal analysis, Data curation, Conceptualization. **Maria Jose Bonta:** Writing – review & editing, Writing – original draft, Visualization, Validation, Resources, Methodology, Investigation, Formal analysis, Data curation, Conceptualization. **Ekaterina Mikhaylova:** Writing – review & editing. **Max Ahnen:** Writing – review & editing. **Simon R. Cherry:** Writing – review & editing, Conceptualization. **Ilaria Sacco:** Writing – review & editing, Supervision, Project administration, Funding acquisition. **Jannis Fischer:** Funding acquisition.

## Declaration of Generative AI and AI-assisted technologies in the writing process

Statement: During the preparation of this work the authors used Google Gemini in order to draft the Abstract and Conclusion sections. After using this tool, the authors reviewed and edited the content as needed and take full responsibility for the content of the publication.

## Declaration of competing interest

The authors declare the following financial interests/personal relationships which may be considered as potential competing interests: Markus Jehl reports a relationship with Positrigo AG that includes: employment and equity or stocks. Maria Jose Bonta reports a relationship with Positrigo AG that includes: employment. Ekaterina Mikhaylova reports a relationship with Positrigo AG that includes: employment and equity or stocks. Max Ahnen reports a relationship with Positrigo AG that includes: employment and equity or stocks. Simon R. Cherry reports a relationship with Positrigo AG that includes: equity or stocks. Ilaria Sacco reports a relationship with Positrigo AG that includes: employment and equity or stocks. Jannis Fischer reports a relationship with Positrigo AG that includes: employment and equity or stocks. If there are other authors, they declare that they have no known competing financial interests or personal relationships that could have appeared to influence the work reported in this paper.

## Footnotes

1 An alternative hardware-based approach tested in time-of-flight (TOF) full-body PET exploits the intrinsic radioactivity of lutetium-based scintillators for attenuation correction [1], [2]. This method requires a large scintillator mass and TOF capability, making it unsuitable for brain-only PET systems, which are the focus of this review.
